# Adverse events of targeted therapies reported by patients with cancer treated in primary care

**DOI:** 10.1080/13814788.2020.1846713

**Published:** 2021-01-05

**Authors:** Samuel Roger, Julien Edeline, Boris Campillo-Gimenez, Elodie Ventroux, Marie-Eve Rouge-Bugat, Anthony Chapron

**Affiliations:** a Department of General Practice, University Rennes, Rennes, France; b CHU Rennes, INSERM, CIC 1414 (Centre d’investigation clinique de Rennes), University Rennes, Rennes, France; c Department of Medical Oncology, Centre Eugène Marquis, Rennes, France; d INSERM, LTSI U1099, Rennes, France; e Clinical Research Department, Centre Eugene Marquis, Rennes, France; f Department of General Practice, University Toulouse, Toulouse, France

**Keywords:** Adverse effect, ambulatory monitoring, cancer survivors, questionnaire, targeted therapies

## Abstract

**Background:**

Targeted Therapies (TT) are among the therapeutic innovations for cancer treatment in outpatient settings. TT-related Adverse Events (AEs) are a source of loss of opportunity for patients if their management is inappropriate.

**Objectives:**

The objective of this study was to describe the AE frequency and severity as reported by patients with cancer who received TT in ambulatory settings. A second objective was to describe the role of the general practitioner (GP) in the management of AEs.

**Methods:**

All patients who started TT at a French Regional Cancer Centre in 2017–2018 were eligible for this 12-month prospective study. A self-administered questionnaire was distributed at inclusion and returned after three months. In the questionnaire, patients listed all AEs that occurred during this period and rated their severity. Occurrence and severity were compared with the rating by a specialised nurse. Patients also indicated the health professional they contacted first for the reported AE.

**Results:**

Among the 247 eligible patients, 15 were excluded and 144 responded to the questionnaire. Fourteen different TTs have been prescribed. Asthenia (92.4%) and anorexia (64.6%) were the most frequent AE. Patients’ AE severity rating was more severe than the nurse’s rating for all drugs (*p* < 0.001). Patients first contacted their GP for 15.6% of AEs, whereas 20.7% of AEs were not reported to any health professional.

**Conclusion:**

Patients experienced an average of 4 AEs. AE severity rating was significantly different between patients and nurses. Patients do not always communicate AEs to health care professionals.

## Introduction

KEY MESSAGES

- Patients with cancer receiving targeted therapy in outpatient settings most frequently reported asthenia and anorexia as adverse effects
- Nurses systematically rated adverse events less severely than patients did
- About one-fifth of the adverse events reported in the patient questionnaire was not communicated to any health professional

The 2018 World Health Organisation (WHO) report on ‘The Health of Europe’ indicates that overall, 2.4% of the population in the 53 ‘European’ countries is currently treated for cancer. Moreover, tumour incidence hugely varies among countries and for the different cancer types [1]. To take care of these patients, healthcare systems are undergoing major changes in Europe: patient education is more often delivered by specialised nurses [2], patients’ empowerment is being promoted [3], electronic applications to improve data collection on adverse events (AE) by patients are increasingly being used [4], and support for AE self-management by the patients is developing fast [5]. Among anticancer treatments, ‘targeted therapies (TT)’ are expanding rapidly. These compounds specifically target a protein or a molecular mechanism implicated in cancer cell proliferation, for instance, a receptor or a growth factor [6]. Their objectives are to improve cancer control and to reduce hospitalisations for cancer treatment [7].

The outpatient delivery or oral administration of some TTs modifies the relationship between compliance, AE management at home, and risk perception by the treated patients and caregivers [8]. In 2012, a primary care study showed an increase in the demand for care by these patients [9]. In this context, Clinical Nurses (CNs) have a major role in AE early identification and therapeutic adaptation, if required [10,11]. These nurses are specifically trained in the management and follow-up of patients with cancer.

To improve the management of patients with cancer, it is also essential to use patient-reported-outcome measurements (PROMs), for instance, self-report questionnaires, to obtain ‘measurements of any aspect of a patient’s health status that come directly from the patient’ [12]. In oncology, several studies, most of which in the context of chemo-radiotherapy, have already shown that PROMs can improve the patient–physician communication [13] as well as symptom management and patient satisfaction [14]. The use of PROMs during TTs has been already evaluated, but only for specific cancers [15–17]. We did not find any study on the evaluation of TT-linked AEs using PROMs. However, the use of PROMs for AE identification and rating, and the patients’ contribution to the organisation of healthcare use can help to improve the quality of life during treatment.

Therefore, the objective of this study was to describe the AEs as reported by patients with cancer receiving TT in outpatient setting, their severity (as rated by the patients and the clinical nurses), and the primary care modalities sought by patients in the event of AEs.

## Methods

### Study design and population

This prospective study was carried out at the Regional Cancer Control Centre (RCCC) of Britany from October 2017 to November 2018. The study was systematically introduced by the CNs to all patients with cancer at the initial consultation for TT in ambulatory setting, regardless of the indication or TT type. Patients with a Performance Status (PS) score rated at inclusion by the oncologist greater than 3 (0–good health to 5–death scale) or with impaired comprehension of written or oral French were not included. Patients who did not express objection to their data collection and analysis and who completed the 3-month follow-up questionnaire constituted the analysed population.

### Cancer management and follow-up

The indication for TT treatment was discussed at a Multidisciplinary Team Meeting (MTM), regardless of the patients’ eligibility for this study. After a consultation for treatment initiation with the medical oncologist, patients were referred to the CNs who is in charge of monitoring all patients treated by TT since 2011. CNs’ roles in the follow-up of patients on TT have been previously described [11]. Briefly, they assess the patient’s understanding of the treatment during an initial consultation, and then during the follow-up consultations (i.e. main principles of TT treatment, follow-up procedures, main AEs and ways to prevent them). Moreover, they monitor by weekly telephone calls the occurrence of TT-related AEs and direct patients to the appropriate healthcare professional in the event of an AE. In the case of AE occurrence, this information was recorded and the AE severity rated by the CN using the French version of the National Cancer Institute – Common Terminology Criteria for Adverse Events (NCI-CTCAE; v4.0) [18].

### Self-monitoring questionnaire and data collected

At the time of the initial consultation with the CN, participants received a follow-up self-administered questionnaire in which they listed all AEs they experienced during the first three months of treatment. At the end of the 3 months, patients returned the questionnaire to the RCCC. If the questionnaire was not received, patients were contacted by telephone by the principal investigator, at most twice. After that, if the questionnaire was not returned, the patient was considered as non-responder. Investigators completed the partially filled in questionnaires together with the patients during a telephone interview within 2 weeks after its reception.

A self-administered questionnaire with 30 questions (Supplementary Appendix 1) was specifically created for this study, based on literature. Four physicians from three different specialties and two CNs validated this questionnaire. It was pre-tested by three patients receiving TT at the Britany RCCC and followed by the CNs (not included in the analysis). This allowed us to assess the excellent understanding of the vocabulary used in the questionnaire, to confirm that the symptoms listed covered the AEs habitually experienced by patients on TT, and to confirm that patients could fill it in on their own.

The questionnaire lists the main AEs of the prescribed TTs, coded using the Medical Dictionary for Regulatory Activities (MedDRA) terminology to transcribe the Patient-Reported Outcome (PRO)-CTCAE terminology criteria into a language that can be understood by the patients [19]. The information collected concerned: AE type, frequency, intensity of the perceived symptoms, and health professional contacted first to manage this problem.

AEs that were not perceived by the patient, but were detected using a laboratory analysis or a measuring device, such as a blood pressure measurement, were not included in this study, which was only interested in what was reported by the patient him/herself. Tumour characteristics and patients’ demographic data were collected retrospectively from the Britany RCCC medical records. Patients rated in the questionnaire their general physical health status and quality of life using a validated 1 to 7 points scale (Supplementary Appendix 1).

The severity of every AE reported by patients in the questionnaire and by the CNs during the regular telephone follow-up was rated from 0 (no AE) to 4 (very severe AE; Supplementary Appendix 1). The highest grade for every AE over the 3 months for the CN and on the questionnaire for the patient was compared. Data were collected in electronic case report forms.

### Statistical analysis

To assess the impact of selection bias related to non-response, the characteristics of responders and non-responders were compared. Categorical variables were compared with the chi^2^ tests or Fisher’s exact tests if applicable; continuous or ordinal variables with the Student’s *t* tests or the Mann–Whitney *U* tests. AE rating by CNs and patients were compared with the Student’s *t* test for matched series or the Wilcoxon signed-rank test if applicable. The agreement between CNs and patients was measured using the weighted Kappa coefficient to take into account the ordinal nature of the NCI-CTCAE grades. Analyses were conducted accepting a 0.05 level of significance. There was no imputation of missing data.

### Ethical and regulatory considerations

The study was approved by the local ethics committee (Opinion N^o^. 17.66, CHU de Rennes [2786]; Supplementary Appendix 2) and was authorised by the French National Commission on Data Protection (CNIL; authorisation No. 2118390 – Supplementary Appendix 3). The study was performed in accordance with the principles of the Helsinki Declaration.

## Results

### Study population

**Figure 1.**
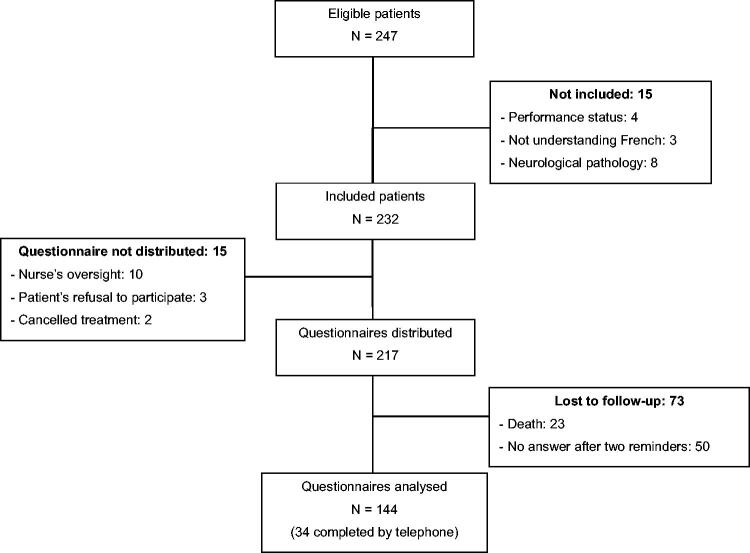
Flowchart.

Between 30 October 2017 and 12 November 2018, 247 patients started TT at the Britany RCCC. Fifteen patients were excluded and 15 did not receive the questionnaire because they refused to participate, the questionnaire was not distributed, or the referring oncologist cancelled treatment after the MTM (Figure 1). In total, 217 patients received the questionnaire. After 3 months, 23 patients had passed away, and 50 did not return the questionnaire despite the two telephone reminders. Finally, 144 questionnaires could be included in the analysis: 110 questionnaires completed by the patients on their own, and 34 questionnaires (23.6%) completed by the principal investigator during a telephone interview.

Women were more inclined to respond and so were patients receiving first line TT. Breast cancer was by far the most common malignancy in both groups. In total, 14 different TT drugs were prescribed, and the most frequent was palbociclib (47.5%) that is indicated for the treatment of breast cancer.

### General patient-reported outcome measures

The performance status score was lower in responders than in non-responders (*p* = 0.05; Table 1). The mean (± SD) scores of the self-evaluated physical health status and quality of life were 4.3 ± 1.43 and 4.4 ± 1.51, respectively, on 1–7 point scales (1 = very poor to 7 = excellent).

**Table 1.** Demographic, medical and therapeutic characteristics.

	Source population (*n* = 217)	Analysed population (*n* = 144)	*p* Value
Sex (women), n (%)	163 (75.1)	114 (79.2)	0.06
Average age (±SD)	62.4 (±11.2)	62.8 (±11)	0.33
Cancer localisation, n (%)			0.06
Breast	122 (56.2)	90 (62.5)	
Kidney	25 (11.5)	12 (8.3)	
Hepatocellular carcinoma	23 (10.6)	10 (7)	
Melanoma	17 (7.8)	12 (8.3)	
Ovary	13 (6)	12 (8.3)	
Sarcoma	6 (2.8)	3 (2.1)	
Thyroid gland	5 (2.3)	3 (2.1)	
Neuroendocrine tumours	4 (1.8)	1 (0.7)	
Cerebral tumour	1 (0.5)	1 (0.7)	
Colon	1 (0.5)	0	
Therapy line, n (%)			*0.02*
First	193 (88.9)	134 (93.1)	
Second	24 (11.1)	10 (6.9)	
Targeted-therapy, n (%)			0.05
Palbociclib	103 (47.5)	73 (50.7)	
TKI multi-targeted	50 (23)	27 (18.8)	
Sorafenib – Sunitinib – Pazopanib – Regorafenib – Lenvatinib			
mTOR inhibitor	22 (10.1)	14 (9.7)	
Everolimus			
EGFR	5 (2.3)	4 (2.8)	
Lapatinib			
VEGFR	8 (3.7)	2 (1.4)	
Vandetanib – Cabozantinib			
PARP inhibitor	14 (6.5)	12 (8.3)	
Olaparib – Niraparib			
Other	15 (6.9)	12 (8.3)	
Dabrafenib – Vemurafenib			
Associated treatment – yes, n (%)	118 (54.4)	86 (59.7)	0.06
Self-evaluated physical health^a^, m (±SD)		4.3 (±1.43)	
Self-evaluated quality of life^a^, m (±SD)		4.4 (±1.51)	
Performance status score, n (%)			0.05
0	81 (37.3)	60 (41.7)	
1	105 (48.4)	67 (46.5)	
2 and more	31 (14.3)	17 (11.8)	

SD: standard deviation; TKI: tyrosine kinase iInhibitor; mTOR: mechanistic target of rapamycin; EGFR: epidermal growth factor receptor; VEGFR: vascular endothelial growth factor receptor; PARP: poly-ADP-ribose polymerase.

a The self-evaluated is a 1–7 scale (1 = very poor to 7 = excellent).

Values in italics indicate values that are statistically significant.

### AEs and evaluation of their severity

A total of 570 AEs were reported by 144 patients, an average of 4 AEs per patient; and 367 AEs were reported by CNs, an average of 2.5 AEs per CN. The CN consistently reports fewer AEs than the patient. The AEs most frequently recorded in the questionnaire by patients were asthenia (92.4%), anorexia (64.6%) and dyspnoea (49.3%). Fever was the least frequent AE both in the patients’ and CNs’ reports (Table 2). The order of frequency of AEs noted by patients and CNs was similar, except for diarrhoea.

**Table 2.** Frequency of adverse events (*N* = 144).

Adverse events	Patient	Clinical nurse
	n (%)	n (%)
Asthenia	133 (92.4)	106 (73.6)
Anorexia	93 (64.6)	57 (39.6)
Dyspnoea	71 (49.3)	50 (34.7)
Nausea	61 (42.4)	37 (25.7)
Diarrhoea	54 (37.5)	27 (18.8)
Mucositis	50 (34.7)	32 (22.2)
Rash	44 (30.6)	29 (20.1)
Hand foot syndrome	34 (23.6)	18 (12.5)
Fever	30 (20.8)	11 (7.6)
Total	570	367

AE severity rating by patients on 0–4 scale (0 = no AE to 4 = very severe AE) was consistently higher than by CNs during the routine follow-up (Table 3). For instance, the mean perceived severity by the patients and CNs was 2.28 and 0.93 for asthenia and 1.25 and 0.49 for anorexia. The order of frequency of AEs was similar except for rash (higher-ranked by patient) and nausea (higher-ranked by nurse). The AE severity rating agreement between patients and CNs was assessed using the Kappa coefficient. The kappa coefficients were generally low, the best three being mucositis 36%, diarrhoea 36% and nausea 35%.

**Table 3.** Comparison of adverse effect grading by clinical nurses and patients.

Adverse effect	Average grade^a^ by patient	Average grade^a^ by nurse	*p* Value	Average of differences [95% CI]	Correlation of nurse–patient grading (Kappa coeff.)
Asthenia, *n* = 133	2.28 (±1.01)	0.94 (±0.68)	< 0.0001	1.34 [1.16; 1.52]	3%
Anorexia, *n* = 93	1.25 (±1.19)	0.49 (±0.68)	< 0.0001	0.77 [0.59; 0.92]	22%
Dyspnoea, *n* = 71	1.17 (±1.36)	0.40 (±0.60)	< 0.0001	0.76 [0.54; 0.99]	13%
Rash, *n* = 44	0.74 (±1.27)	0.23 (±0.48)	< 0.0001	0.51 [0.30; 0.73]	7%
Mucositis, *n* = 50	0.57 (±0.97)	0.26 (±0.53)	< 0.0001	0.31 [0.18; 0.43]	36%
Nausea, *n* = 61	0.55 (±0.79)	0.31 (±0.60)	< 0.0001	0.26 [0.14; 0.38]	35%
Diarrhoea, *n* = 54	0.52 (±0.82)	0.22 (±0.48)	< 0.0001	0.31 [0.19; 0.42]	36%
Hand foot syndrome, *n* = 34	0.39 (±0.79)	0.17 (±0.51)	0.0004	0.22 [0.10; 0.34]	29%
Fever, *n* = 30	0.27 (±0.57)	0.08 (±0.30)	0.0002	0.19 [0.09; 0.28]	16%

^a^Mean ± standard deviation.

CI: confidence interval; HFS: hand-foot syndrome.

### AE management

In the case of AE, most patients first contacted the CN (34.6% of AEs) and then their GP (15.6%) or oncologist (14.9%). However, 20.7% of AEs recorded in the questionnaire were not reported to any health professional (Table 4). The most frequent AE not sought advice were asthenia, anorexia and dyspnoea; and the most severe grading, relatively, were asthenia (6/14) and rash (4/14) graded 3 or 4.

**Table 4.** Adverse event rating by patients who did not seek advice for that adverse event.

*N* = 118	number of adverse events for which no advice was sought	Grading			
		1	2	3	4
Asthenia	30	6	18	5	1
Anorexia	21	15	5	1	
Dyspnoea	16	4	10	1	1
Diarrhoea	13	11	1	1	
Nausea	9	9			
Hand foot syndrome	8	8			
Mucitis	8	8			
Rash	7	3		4	
Fever	6	4	2		
TOTAL	118	68	36	12	2
%		57.7	30.5	10.2	1.6

## Discussion

### Main findings

In this study, 144 of 232 patients who started TT in the Brittany RCCC, rated quality of life at 4.4 (1–7 scale) and self-reported physical health at 4.3 (1–7 scale). Performance status was 0 or 1 for 85% of patients (0–4 scale), meaning that their state of health was quite suitable for outpatient treatment with some minor activity restrictions. Of the 144 patients who completed an adverse event questionnaire for 3 months, the total number of patient-reported adverse events was 570, with an average of 4 AEs per patient. Nurses consistently reported fewer AEs (mean 2.5 per CN). The most frequently reported AEs were asthenia, anorexia and dyspnoea. Agreement on the severity of AEs between patients and nurses was low (kappa 7–36%). Not for all adverse events professional help was sought (20.7%). The most severe unreported AEs were asthenia and rash.

### Strengths and limitations

This study is the first to assess AE frequency and severity by using a PROM in patients with cancer receiving oral TT in an outpatient setting. 58% of patients filled in and sent back the questionnaire and 76.4% of them answered all the questions. AE occurrence was precisely reported by patients in the self-administered questionnaire (19). The validated PRO-CTCAE questionnaire, translated into French in 2018, was not available at the start of our study [20].

However, our study concerned only one RCCC. A multicentre study would be useful to enlarge the list and number of prescribed TTs and to analyse other AEs and cancer management practices.

The AE types included in the self-administered questionnaire and the focus on the first 3 months of treatment were decided on the basis of the results of pharmacovigilance studies on the most commonly used TTs [11,21]. Moreover, to increase the participation rate, it was decided to finalise some incomplete questionnaires by telephone interview. This might have introduced an information bias due to the use of two different techniques for data collection. Specifically, some patients could have forgotten some AEs they experienced (recall bias), or their answers could have been influenced by the direct contact with a physician (social desirability bias).

#### Reported AEs

The AEs reported in this study are those usually observed with the TTs prescribed to the included patients [22]. The most frequently reported AEs were subjective symptoms, such as asthenia, anorexia, and dyspnoea. Asthenia was listed by 92% of patients, as previously observed in patients receiving chemotherapy and radiotherapy (>90%) [23,24].

### AE severity rating discrepancy between nurse and patient

Subjective symptoms are challenging to qualify and quantify by nurses and physicians during follow-up consultations. Moreover, the AE rating scales developed to improve reproducibility in clinical research, favour their quantitative evaluation. Conversely, patients describe AE severity according to their perception, thus giving a subjective assessment, and this might lead to discrepancies [25]. Indeed, the patients’ ratings of TT-related AEs were higher than those by CNs, as previously reported in a study on patients with breast cancer treated with adjuvant chemotherapy [26]. Overall, health professionals tend to underestimate the severity of AEs, possibly because they focus mostly on life-threatening AEs, and neglect some AEs that may bring much suffering to patients [27]. It is precisely this subjectivity that is currently exploited in PRO questionnaires to improve symptom monitoring and management. For instance, the Symptom Tracking and Reporting (STaR) web-based interface in advanced solid tumours and Moovcare^®^ [4,17], a digital questionnaire to detect relapse/complications during lung cancer follow-up, gave convincing results on quality of life and overall survival compared to standard follow-up according to current recommendations.

### Healthcare service use in the event of AEs

The healthcare professional most often called in the case of AE was the CN (34.6% of AEs), possibly due to their weekly contact by telephone with the patients. Nevertheless, about 30% of AEs were first reported to the GP or oncologist, despite the CN’s regular follow-up. This could be explained by the appearance of the AE after the end of the regular CN’s follow-up. Moreover, in France, the care pathway is coordinated by the patient’s GP, and in our study, all patients had a GP. Therefore, patients may feel more at ease, may trust more their GP, or may think that their GP can better deal with this kind of symptoms. A qualitative study could allow the identification of the factors underlying these choices. We might also ask whether the questions asked by the CN are really adapted to understand the patient’s status fully. Indeed, in oncology, data collection systems are mainly based on objective criteria that are not adapted to assess the patient’s subjectivity who will then look for another professional in the hope to be better understood.

Nevertheless, this patient behaviour shows that primary care professionals have a role in the follow-up of patients, in addition to the follow-up by the cancer centre [28].

Moreover, almost 21% of the AEs listed by patients were not reported to any health professional. This might be explained by the low severity (grade 1) of most of them (Table 4) and consequently, their minor influence on the patient’s daily life. However, fear of a treatment change (dose reduction, treatment pause or discontinuation) could be another reason and it be related to the phenomenon of over-observance [8], which is often observed in patients with cancer [29]. A qualitative study of our population would be useful to explore this attitude.

### Implications for clinical practice

The use of a PROM allows for a comprehensive collection of AEs and a better appreciation of how patients experience those, avoiding underestimation of how they feel [30], as would better communication between GPs, specialist nurses and oncologists avert the loss of information, as we have noted [31]. For example, lists of AEs specific to ongoing TT could be sent by the oncologist to the GP to support primary care management [32]. Or some GPs could participate in team meetings to ensure continuity between the oncologist and follow-up in primary care [33,34].

In a context of increasing prescription of TTs and to enable GPs to react better to AEs these treatments, specific initial and continuous education would enable them to manage better in their daily practice [35].

For optimal management of AEs by the patient at home, therapeutic education sessions could be organised to improve knowledge of AEs and their management [11,36].

## Conclusion

Asthenia, anorexia and dyspnoea are the AEs most frequently reported by patients undergoing treatment with oral TTs. A significant proportion of AEs self-reported by patients are not shared with any health care professional. Our study also highlighted the significant difference in AE rating between CNs and patients, showing that the current quantitative scales used by CNs underestimate patients’ perceptions. Finally, our study suggests that GPs have a meaningful and complementary role in the follow-up of patients with cancer.

## Supplementary Material

Supplemental Material: Patient Questionnaire
