# Supplementary figures and images for: Adverse events of targeted therapies reported by patients with cancer treated in primary care
Source: Eur J Gen Pract. 2021 Jan 5;26(1):202–9. doi: 10.1080/13814788.2020.1846713 (PMC7801082; doi:10.1080/13814788.2020.1846713)

**
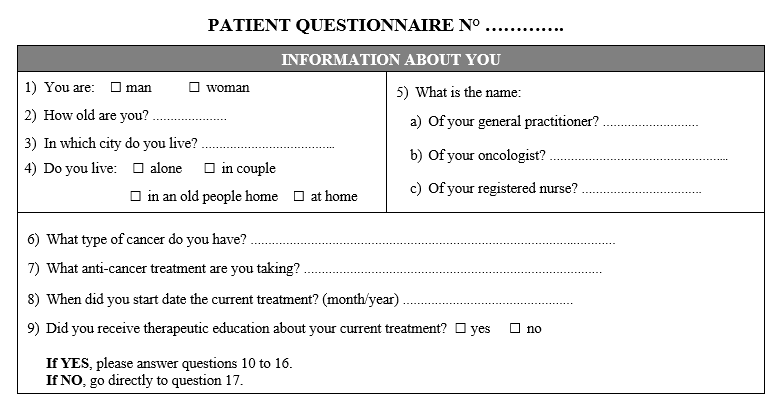
PATIENT QUESTIONNAIRE**


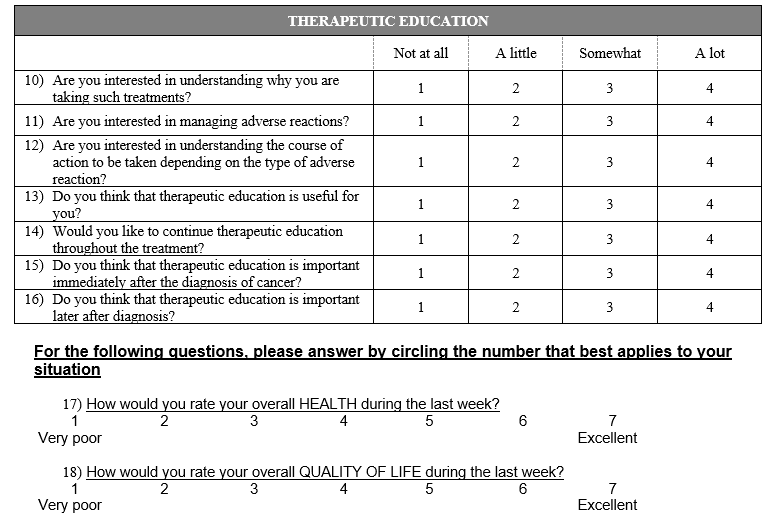


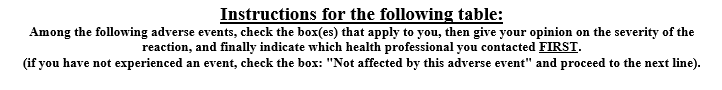
**
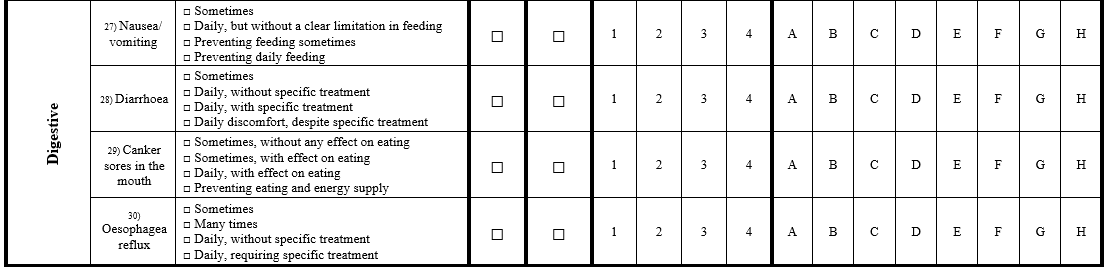
**
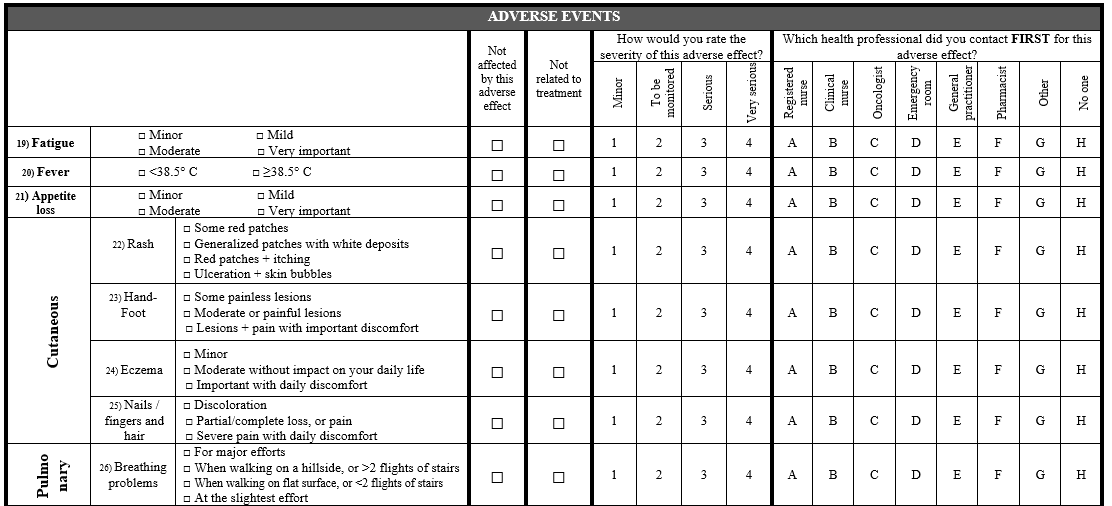

Supplement: Supplemental Material: Patient Questionnaire [file IGEN_A_1846713_SM9177.docx]
